# Descriptive analysis of horses and ponies attending horse auctions in Victoria from July 2019 to March 2020

**DOI:** 10.1111/avj.13210

**Published:** 2022-09-29

**Authors:** GR Chapman, B Wells, JR Gilkerson, ML Flash

**Affiliations:** ^1^ Hastings Victoria Australia; ^2^ Bairnsdale Victoria Australia; ^3^ Asia‐Pacific Centre for Animal Health, Faculty of Veterinary and Agricultural Sciences The University of Melbourne Parkville Victoria Australia

**Keywords:** horse, sale, saleyard, standardbred, thoroughbred, welfare

## Abstract

**Introduction:**

In recent years there has been public speculation about the breed, destination and number of horses being sold by public auction at livestock saleyards in Australia. Currently, there is little objective information available about the breed and condition of horses sold through this medium. With little publicly available objective data on these horses, the horse industry has been left vulnerable to misinformation. Accurate information regarding the composition and condition of horses attending saleyards is important to identify and address any welfare issues and to inform public debate.

**Method:**

Data were collected on 312 horses and ponies presented for sale through the Pakenham Horse Sales between July 2019 and March 2020. All horses and ponies were inspected at the saleyards and information on breed, age, body condition score (BCS), purchaser and sale price were recorded as the animals were auctioned.

**Results:**

Crossbred horses and ponies were the largest groups presented for sale. Ponies were more likely to be sold to private buyers. Quarter horses and riding ponies were as likely to be sent to slaughter as thoroughbreds and standardbreds. Entire males and females sold for lower prices than geldings. Most horses and ponies (64%) were sold to private buyers. More than three‐quarters (77%) of horses and ponies presented for sale had a BCS greater than or equal to three out of five.

**Conclusion:**

This pilot study challenges perceptions that thoroughbreds are the primary breed to attend public sales or that animals attending the sales are in poor condition.

In recent years there has been an increased focus by the community, political parties and animal welfare organisations on the number of thoroughbred and standardbred horses being sent to the slaughterhouse.^1^, ^2^, ^3^, ^4^, ^5^, ^6^, ^7^, ^8^ The increased interest, together with a lack of reliable information on the composition and condition of horses that attend saleyards in Australia, has led to a range of opinions about the outcomes for these horses.^1^, ^2^, ^3^, ^4^, ^5^, ^6^, ^7^, ^8^ Although other horse breeds are also being sent to slaughterhouses there is currently little known about the number of horses and by which route they are being processed.^1^, ^2^, ^8^, ^9^, ^10^, ^11^, ^12^, ^13^ A 2001 report on the Australian horse industry concluded there was little information available on the sale of horses other than thoroughbreds.^14^

Previous research has indicated that recreational horse owners primarily buy and sell horses and ponies through private sales, only 2% of recreational owners reported that they use knackeries, and 3% sell through auctions/saleyards to dispose of horses or ponies when they are no longer needed.^15^ The same study reported that only 2% of horses owned by recreational horse owners were acquired through a saleyard and 4% through a dealer.^15^

There is a perception amongst the public, animal welfare and rights organisations, and the media that majority of horses being sent to slaughter are thoroughbreds.^4^, ^5^, ^6^, ^9^, ^11^ There is also the perception that the majority of horses being sold at horse sales end up being sent to slaughter, although there have been no data available previously to support or refute these claims.^16^

The aim of this pilot study is to begin to fill the gaps in our knowledge about the number, type, breed and condition of horses and ponies attending public sales at the Pakenham Livestock Exchange. The sale price and purchaser were also recorded. The results of this study will provide insight into the numbers of horses and ponies being sold through this venue, the number of animals being sold to slaughterhouses, and an estimate of the age, body condition and breeds of the horses that are presented for sale. Our hypothesis was that thoroughbreds were not the majority breed group that attended the sales, nor were they the largest breed group that was purchased by slaughterhouse buyers.

## Methods

### Study population

All horses presented for sale by Pakenham Horse Auctions at the Pakenham Livestock Exchange, from July 2019 to March 2020, were included in the study population to determine the total number, breed, sex and ages of the horses sold in this period. Sales were held once per month, with no sales held in January 2020. The sales ceased and data collection was suspended in April 2020 due to COVID‐19 restrictions. One researcher, with training in body condition scoring and written instructions, attended all eight sales to observe the horses and ponies, and collect data. Data were collected in two sets during each sale. The first set was collected prior to the auction and recorded the date of sale, pen number and horse details. The second set was collected during the auction and recorded the pen number, purchaser details, whether the horse was sold and purchase price. The two sets were matched using the pen number of the horse.

The outcome of the sale was recorded, including the type of purchaser and purchase price in Australian dollars. The type of purchaser was categorised into four groups: buyers who were known to purchase horses to be processed at abattoirs or knackeries (slaughterhouse), buyers who on‐sell horses to third‐party private or commercial buyers (dealer), private buyers (private) or rescue organisations (rescue). The purchaser descriptions and subsequent categorisation were based on sale records and local business knowledge. Horses (n = 4, two Crossbred horses, one Paint horse, one thoroughbred) sold to ‘dealers’ were included in the ‘slaughterhouse’ (n = 107) category because some dealers on‐sell horses to slaughterhouses if they cannot find a private buyer to purchase the horse; ‘slaughterhouse’ and ‘dealer’ buyers are reported under the collective heading of ‘slaughterhouse’ in the table but are detailed separately in the text. Rescue organisations (n = 2) were included with ‘private’ buyers (n = 192), because they were unlikely to be sold for slaughter, for reporting in the tables but are detailed separately in the text.

Information on the horse breed, age and sex; details of body condition and hoof condition were also recorded. Microchip, registration and/or names were recorded if they were provided on the vendor declaration form. Brands were photographed. Individuals were categorised as ponies if they were at or below a height of 144 cm (14.2 hands high), measured from the ground to the top of the wither. Horses were those that measured greater than 144 cm high. Horse sex was determined from information provided by the vendor, information provided by the auctioneer, or after visual examination. ‘Females’ were all fillies and mares. ‘Males’ were any male horses attending the sales. Males were further sub‐classified as ‘Geldings’ if they had been castrated and ‘Entire male’ if they had not been castrated. The body condition score (BCS) of the horse was graded on a scale from 0 to 5.^17^ Hoof condition was graded as ‘Poor’, ‘Okay’ and ‘Good’. Hoof condition was considered ‘Poor’ if there were large cracks or chips in the hoof wall, or if the hoof was excessive in length. If there was evidence of regular or semi‐regular care that may not have been undertaken recently, they were categorised as ‘Okay’. Horses categorised as having ‘Good’ hoof condition had hooves that were neatly shaped and appeared to have recently received attention.

Horses were observed from all sides from the outside of the pen, to determine sex of horse, body condition and hoof condition. Researchers did not enter the pens with the horses for safety reasons. Age was broadly categorised as immature, young, mature, aged and unknown. Age was estimated using the visual appearance of the horse, presence of brands, and/or age on the information provided by the vendor or auctioneer. The visual inspection included identifying the equine as a foal or very young animal (immature), and conversely the presence of grey hair and other signs of advanced age (aged). Horses and ponies of foal up to 12 months of age were categorised as ‘Immature’. ‘Young’ horses and ponies were those aged 12 months to 4‐years. ‘Mature’ horses and ponies were those aged 5 to 16 years. Horses and ponies aged 17 years and over were categorised as ‘Aged’.

Information provided by the vendor or auctioneer, the presence of brands and visual inspection were used to determine the breed of the horse. ‘Crossbred’ refers to horses and ponies were the breed information, included two or more breeds. This applies to both horses and ponies. Horse breeds with fewer than 10 individuals were categorised as other – horse or other – pony as appropriate. Appaloosa (n = 6), Paint (n = 7), Arab (n = 2), Clydesdale (n = 1), Warmblood (n = 1) were collapsed into ‘other – horse’. The breeds Brumby (n = 3), Hackney (n = 1), Miniature (n = 3) and Welsh pony (n = 7) were collapsed into ‘other – pony’.

### Data analysis

Continuous (number of horses/ponies, sale price) and categorical variables (breed, sex, age, BCS, hoof condition, sale outcome, price) were compared using t‐tests and chi‐squared tests, respectively. Non‐normally distributed data (sale price) was log‐transformed prior to analysis. Percentages were calculated by dividing the number of horses and/or ponies of interest by the total number of study horses and ponies (n = 312), unless otherwise indicated. Analysis of variance was used to analyse the difference in sale price between breeds and Tukey's honestly significant difference test was used to compare the sale price of each breed category.

## Results

There were 312 horses and ponies sold at eight sales between July 2019 and March 2020. The median number of horses and ponies to attend each sale was 33 (Quartile 1 [Q1] 26, Quartile 3 [Q3] 43). The lowest number of horses and ponies (5%, 16 of 312) attending a sale occurred in August 2019 and the largest number of horses and ponies (29%, 90 of 312) to attend a sale was in December 2019 (Table 1).

**Table 1:** Breeds of horses and ponies attending Pakenham horse auction between June 2019 and March 2020

	Sale date									
	2019 July	2019 Aug	2019 Sept	2019 Oct	2019 Nov	2019 Dec	2020 Feb	2020 Mar	Total	
Breed	n	n	n	n	n	n	n	n	n (%)	95% CI
Crossbred										
Horse	4	4	5	5	4	5	6	1	34 (11)	8 to 15
Pony	8			12	1	34	5		60 (19)	15 to 24
Subtotal	12	4	5	17	5	39	11	1	94 (30)	25 to 35
Thoroughbred	5	6	7	11	9	3	3	7	51 (16)	13 to 21
Unknown	5		2		1	23	5	1	37 (12)	9 to 16
Quarter horse			3	6		4	2	12	27 (9)	6 to 12
Standardbred	2	1	5	1	1	4	2	4	20 (6)	4 to 10
Riding pony	10	1			3		1	3	18 (6)	4 to 9
Shetland	10	1			2	5			18 (6)	4 to 9
Stock horse	3	1	1	1	1	6		1	14 (4)	3 to 7
Other – horse	1	1		3	2	3	6	2	18 (6)	4 to 9
Other – pony	3	1		1	3	3	3	1	15 (5)	3 to 8
Total n (%)	51 (16)	16 (5)	23 (7)	40 (13)	27 (9)	90 (29)	33 (11)	32 (10)	312 (100)	
95% CI	13 to 21	3 to 8	5 to 11	10 to 17	6 to 12	7 to 13	8 to 14	7 to 14		

	Sale date									
Breed	2019 July	2019 Aug	2019 Sept	2019 Oct	2019 Nov	2019 Dec	2020 Feb	2020 Mar	Total	
	n	n	n	n	n	n	n	n	n (%)	95% CI
Crossbred										
Horse	4	4	5	5	4	5	6	1	34 (11)	8 to 15
Pony	8			12	1	34	5		60 (19)	15 to 24
Subtotal	12	4	5	17	5	39	11	1	94 (30)	25 to 35
Thoroughbred	5	6	7	11	9	3	3	7	51 (16)	13 to 21
Unknown	5		2		1	23	5	1	37 (12)	9 to 16
Quarter horse			3	6		4	2	12	27 (9)	6 to 12
Standardbred	2	1	5	1	1	4	2	4	20 (6)	4 to 10
Riding pony	10	1			3		1	3	18 (6)	4 to 9
Shetland	10	1			2	5			18 (6)	4 to 9
Stock horse	3	1	1	1	1	6		1	14 (4)	3 to 7
Other – horse	1	1		3	2	3	6	2	18 (6)	4 to 9
Other – pony	3	1		1	3	3	3	1	15 (5)	3 to 8
Total n (%)	51 (16)	16 (5)	23 (7)	40 (13)	27 (9)	90 (29)	33 (11)	32 (10)	312 (100)	

	Sale date									
	2019 July	2019 Aug	2019 Sep	2019 Oct	2019 Nov	2019 Dec	2020 Feb	2020 Mar	Total	
Breed	n	n	n	n	n	n	n	n	n (%)	95% CI
Crossbred										
Horse	4	4	5	5	4	5	6	1	34 (11)	8 to 15
Pony	8			12	1	34	5		60 (19)	15 to 24
Subtotal	12	4	5	17	5	39	11	1	94 (30)	25 to 35
Thoroughbred	5	6	7	11	9	3	3	7	51 (16)	13 to 21
Unknown	5		2		1	23	5	1	37 (12)	9 to 16
Quarter horse			3	6		4	2	12	27 (9)	6 to 12
Standardbred	2	1	5	1	1	4	2	4	20 (6)	4 to 10
Riding pony	10	1			3		1	3	18 (6)	4 to 9
Shetland	10	1			2	5			18 (6)	4 to 9
Stock horse	3	1	1	1	1	6		1	14 (4)	3 to 7
Other – horse	1	1		3	2	3	6	2	18 (6)	4 to 9
Other – pony	3	1		1	3	3	3	1	15 (5)	3 to 8
Total n (%)	51 (16)	16 (5)	23 (7)	40 (13)	27 (9)	90 (29)	33 (11)	32 (10)	312 (100)	

Other – horse: Appaloosa (n = 7), Paint (n = 7), Arab (n = 2), Clydesdale (n = 1), Warmblood (n = 1); Other – pony: Brumby (n = 3), Hackney pony (n = 1), Miniature pony (n = 4), Welsh pony (n = 7), CI = Confidence Interval. Abbreviations: Aug = August, Sept = September, Oct = October, Nov = November, Dec = December, Feb = February, Mar = March.

**Figure 1:**
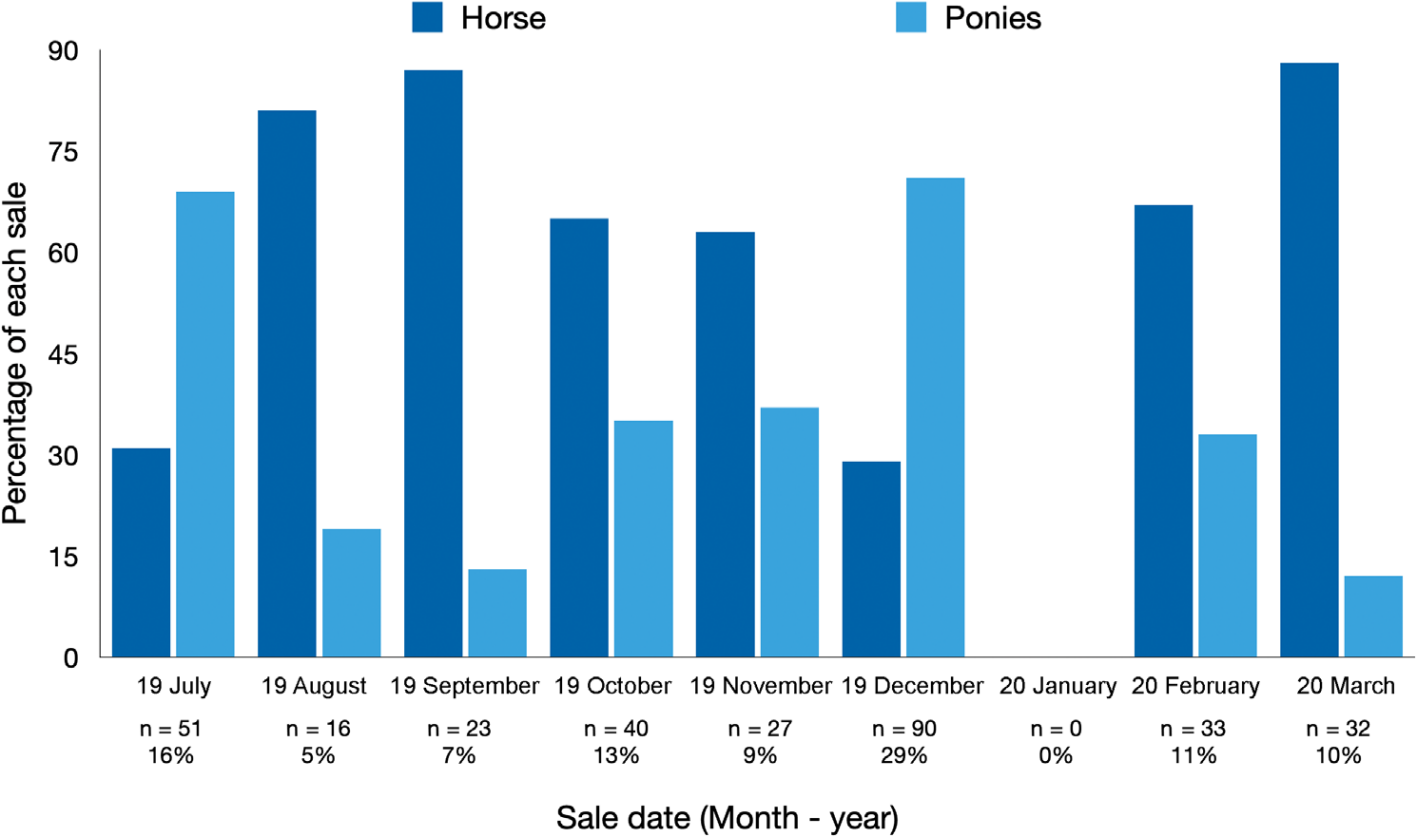
Number of horses and ponies attending Pakenham horse auction between July 2019 and March 2020 (n = 312).

While horses (55%, 170 of 312) were the largest group to attend the sales overall, ponies outnumbered horses at the July 2019 sale (69%, 35 of 51) and the December 2019 sale (70%, 63 of 90, Figure 1). This was partly due to a large group of ponies of unknown breed, that had features and heights similar to the Shetland breed and came from one vendor at the December sale. Five ponies from this consignment were identified as Shetlands and the remainder were recorded as Crossbred–ponies (Table 1).

### Breeds

Overall, crossbred horses and ponies were the most frequent (30%, 94 of 312) breed group that attended the sales (Table 1). Crossbreds were more likely to be ponies (64%, 60 of 94) than horses (36%, 34 of 94, χ^2^ test statistic = 18.20, df = 1, P < 0.001). The most frequent breed description for crossbreds was Shetland crosses (32%, 30 of 94). Thoroughbreds were the second most frequent breed (16%, 51 of 312) attending the sales (Table 1). Quarter horses (9%, 27 of 312), followed by Standardbreds (6%, 20 of 312), were the next two most frequent breeds (Table 1). Ponies (10%, 31 of 312) were more likely than horses (2%, 6 of 312) to be of unknown breeding (χ^2^ test statistic = 24.79, df = 1, P < 0.001).

### Sex and age

Females were the most frequent (54%, 167 of 312) sex to attend the sales, followed in descending order by geldings (34%, n = 106 of 312) and entire males (13%, 39 of 312, Table 2). Entire males attending the sales were more likely to be ponies (64%, n = 25 of 39) as horses (36%, 14 of 39, χ^2^ test statistic = 6.21, df = 1, P = 0.01). Half of all the entire males (49%, 19 of 39) were ponies from one sale (Dec‐19). Horses (42%, 72 of 170) were more likely than ponies (24%, 34 of 142) to be geldings (χ^2^ test statistic = 11.69, df = 1, P < 0.001). There was no significant difference in the number of females between horses (49%, n = 84 of 170) and ponies (58%, 83 of 142), (χ^2^ test statistic = 2.54, df = 1, P = 0.11).

**Table 2:** Number and percentage of age, body condition score and hoof condition categories for horses and ponies attending Pakenham horse auction between June 2019 and March 2020 by sex

	Female	Male	Total
Variable	n (%)	n (%)	n (%)
Age			
Immature	7 (39)	11 (61)	18 (5.8)
Young	31 (52)	30 (48)	61 (20)
Mature	115 (56)	90 (44)	205 (66)
Aged	5 (50)	5 (50)	10 (3.2)
Unknown	9 (50)	9 (50)	18 (5.8)
Body condition score			
One	1 (50)	1 (50)	2 (0.6)
Two	37 (54)	32 (46)	69 (22)
Three	125 (55)	103 (45)	228 (73)
Four	4 (31)	9 (69)	13 (4.2)
Hoof condition			
Poor	45 (61)	29 (39)	74 (24)
Okay	86 (57)	66 (43)	152 (49)
Good	35 (42)	49 (58)	84 (27)
Unknown	1 (50)	1 (50)	2 (0.6)
Total n (%)	167 (54)	145 (46)	312 (100)

Immature = 0–1 years of age, Young = 2–4 years of age, Mature = 5–16 years of age and aged >16 years. Body condition score calculated using Carroll‐Huntington scale of zero to five. Hoof condition: Poor = large cracks or chips in the hoof wall, or if the hoof was excessive in length; Okay = regular or semi‐regular care that may not have been undertaken recently, Good = hooves that were neatly shaped and appeared to have recently received attention.

There was no significant difference between males and females for thoroughbreds (χ^2^ test statistic = 0.27, df = 1, P = 0.60) or Standardbreds (χ^2^ test statistic = 0.02, df = 1, P = 0.89). Females (81%, n = 30 of 37) were more likely than males (19%. n = 7 of 37) to be of unknown breeding (χ^2^ test statistic = 12 0.81, df = 1, P < 0.001).

The majority (66%, 196 of 312) of the horses and ponies attending the sales were a mature age, followed by young, immature and aged with 5.8% (17 of 312) of unknown age (Table 2). Immature and young horses and ponies that attended the sale were more likely to be female (χ^2^ test statistic = 4.454, df = 1, P = 0.04) and entire males (χ^2^ test statistic = 53.22, df = 1, P < 0.001) compared to geldings. There was no difference in the proportion of mature thoroughbreds (77%, 39 of 51, χ^2^ test statistic = 3.14, df = 1, P = 0.08) or mature Standardbreds (70%, 14 of 20, χ^2^ test statistic = 0.175, df = 1, P = 0.68) when compared to the rest of the study population.

### Body condition score

The majority (73%, 219 of 312) of horses and ponies attending the sales had a BCS of three, followed by those with a BCS of two and four (Table 2). Fewer than 1% (0.6%, 2 of 312) of horses and ponies attending the sales had a BCS of one. There were no horses or ponies attending the sales with a BCS of five. Horses (12%, 38 of 312) were no more likely to be presented at the sales with a BCS one or two than ponies (10%, 31 of 312, χ^2^ test statistic = 0.012, df = 1, P = 0.91).

When investigated by breed, thoroughbreds (20%, 14 of 71) were as likely to have a BSC of less than three, compared to other breeds (80%, 57 of 71, χ^2^ test statistic = 0.62, df = 1, P = 0.43, Table 2). Similarly, Standardbreds (8%, 6 of 71) were as likely as the rest of the study horses to have a BCS of less than three (χ^2^ test statistic = 0.64, df = 1, P = 0.42). There was no significant difference between the sexes in the likelihood of having BCS of less than 3 when attending the sales (χ^2^ test statistic = 0.00, df = 1, P = 1.00, Table 2).

### Hoof condition

The majority (76%, 236 of 312) of horses and ponies had hooves that were either in ‘okay’ or ‘good’ condition (Table 2). Horses (30%, 51 of 170) were more likely to have ‘poor’ hoof condition than ponies (16%, 23 of 142, χ^2^ test statistic = 8.15, df = 1, P = 0.001). Males (20% 29 of 145) were as likely as females (27%, 45 of 167) to be presented for sale with hooves in ‘poor’ condition (χ^2^ test statistic = 2.07, df = 1, P = 0.15). When the hoof condition for breed groups was investigated, quarter horses (19%, 14 of 74) were more likely to have ‘poor’ hoof conditions than other breeds (81%, 60 of 74, χ^2^ test statistic = 12.9, df = 1, P < 0.01).

### Sale outcome and purchaser type

The purchaser type and sales price were available for 97% (304 of 312) of horses and ponies presented for sale. Of the eight horses and ponies where the purchaser and/or sale price was unavailable, one was recorded as sold but the purchaser type and price were not available, one was sold to a slaughterhouse and the price was not recorded, one was unsold and five were passed in. These eight horses and ponies were excluded from further analysis.

The majority (64%, 194 of 304) of horses and ponies attending the sales were purchased by private buyers (Table 3). Ponies (56%, 108 of 194) were more likely than horses (44%, 86 of 194) to be purchased by private buyers (χ^2^ test statistic = 21.91, df = 1, P < 0.001, Table 3). Over one‐third of horses and ponies (36%, 110 of 304) sold at the sales were sold to slaughterhouse buyers. Horses (73%, 80 of 110) were more likely than ponies (27%, 30 of 110) to be purchased by slaughterhouse buyers (χ^2^ test statistic = 22.84, df = 1, P < 0.001, Table 3). There was no significant difference between horses and ponies purchased by slaughterhouse vs private buyers when compared by BCS (χ^2^ test statistic = 1.56, df = 3, P = 0.67) or hoof condition (χ^2^ test statistic = 6.488, df = 3, P = 0.09). There was no significant difference between the age categories of the horses and ponies purchased by private and slaughterhouse buyers (χ^2^ test statistic = 6.356, df = 4, P = 0.17). Females (64%, 70 of 110) were more likely than males (36%, 40 of 110) to be purchased by slaughterhouse buyers (χ^2^ test statistic = 5.672, df = 1, P = 0.01). Thoroughbreds and Standardbreds were more likely to be purchased by slaughterhouse buyers (64%, 45 of 70) when compared to non‐racing breeds (28%, 66 of 234) of the horses and ponies attending the sales (χ^2^ test statistic = 30.26, df = 1, P < 0.001). However, there was no significant difference in the likelihood of riding ponies (33%, 6 of 18), quarter horses (42%, 11 of 26) and standardbreds (55%, 11 of 20) being purchased by slaughterhouse buyers when compared to thoroughbreds (68%, 34 of 50, Table 3).

**Table 3:** Purchaser type by breed for horses and ponies attending Pakenham horse auction between June 2019 and March 2020

	Slaughterhouse	Private		Totals
Breed	n (%)	n (%)	P^a^	n (%)
Thoroughbred	34 (68)	16 (32)	Ref	50 (16)
Crossbred–horses	11 (32)	23 (68)	<0.01	34 (11)
Crossbred–ponies	17 (29)	41 (71)	<0.01	58 (19)
Quarter horse	11 (42)	15 (58)	0.37	26 (9)
Standardbred	11 (55)	9 (45)	1.00	20 (7)
Riding pony	6 (33)	12 (67)	0.25	18 (6)
Shetland	3 (17)	14 (82)	<0.01	17 (6)
Stock horse	3 (23)	10 (76)	0.05	13 (4)
Pony – other	2 (14)	12 (85)	<0.01	14 (4)
Horse – other	6 (35)	11 (64)	0.14	17 (6)
Unknown	6 (16)	31 (83)	<0.01	37 (12)
Total n (%)	110 (36)	194 (64)		304 (100)

Other – horse: Appaloosa (n = 6), Paint (n = 7), Arab (n = 2), Clydesdale (n = 1), Warmblood (n = 1); Other – pony: Brumby (n = 3), Hackney pony (n = 1), Miniature pony (n = 3), Welsh pony (n = 7). Excludes – five horses and ponies that were passed in, one each of the following breeds: thoroughbred, quarter horse, stock horse, Pony – other and Horse – other; one pony (Shetland) that was unsold; and two horses and ponies that were sold to an unknown purchaser.

^a^ Differences within breeds were assessed using ANOVA. Tukey's Honestly Significant Difference test was used to compare breeds with the stated reference.

### Sale prices

**Figure 2:**
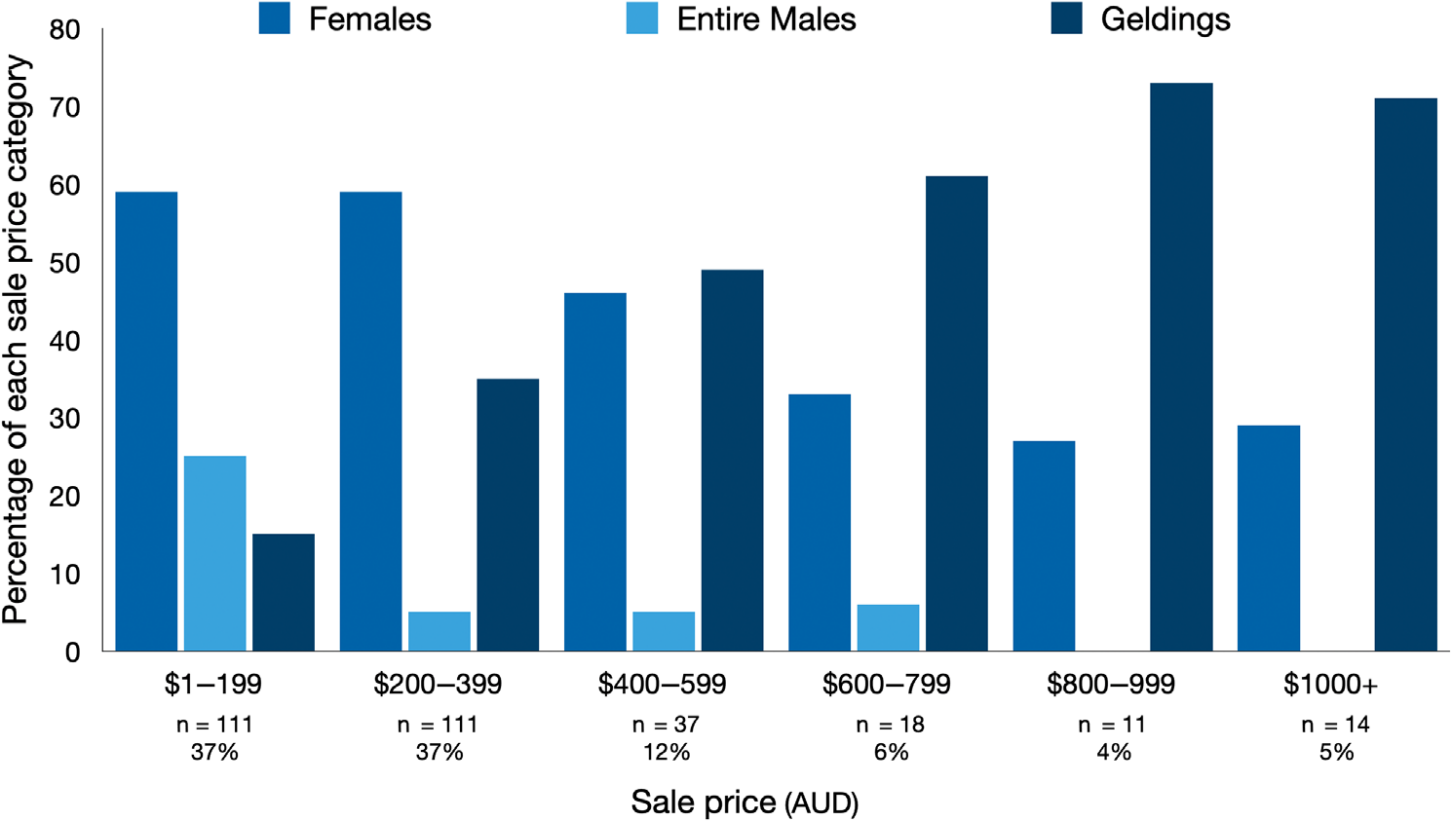
The sales price for horses by sex sold at Pakenham horse auction between July 2019 and March 2020 (n = 302). Excludes‐horses and ponies that were passed in (n = 5) or where the sex was unknown (n = 5).

The median sale price for all horses and ponies sold was $235 (Q1 $130, Q3 $400). Eighty‐five percent of horses and ponies (259 of 304) sold for $500 or less, with nearly a quarter (24%, 74 of 304) sold for between $101 and $200 (Figure 2). Only 4% (13 of 304) sold for more than $1000 (Figure 2).

The median sale price for ponies was $135 (Q1 $80; Q3 $229) which was significantly less than the median sale price for horses of $335 (Q1 $200, Q3 $495), (t‐test statistic 8.07, df = 219.19, P < 0.001). Horses and ponies sold to slaughterhouse buyers (median $200, Q1 $150, Q3 $300) were purchased for lower prices than those sold to private buyers (median $280, Q1 $106, Q3 $480), (t‐test statistic 4.45, df = 299.99, P < 0.001). Entire males (median $105, Q1 $63, Q3 $185, P < 0.01) and females were more likely to be sold at a lower price (median $200, Q1 $100, Q3 $350, P < 0.01) than geldings (median $350, Q1 $225, Q3 $600, Figure 2). There was no significant difference in sale price between breed groups, with the exception of a higher median price for Crossbred horses (Table 4).

**Table 4:** Sale price by breed for horses and ponies attending Pakenham horse auction between June 2019 and March 2020

Breed	Sale price		
	n	Median (IQR)	Min, max	P^a^
Thoroughbred	50	300 (225, 400)	1, 1250	Ref
Crossbred‐horses	34	500 (335, 757)	1, 2100	0.01
Crossbred‐ponies	58	125 (70, 200)	1, 950	0.86
Quarter horse	26	300 (205, 488)	1, 2550	0.18
Standardbred	20	255 (200, 358)	1, 440	1.00
Riding pony	18	100 (73, 243)	1, 680	0.72
Shetland	17	100 (60, 200)	1, 300	0.38
Stockhorse	13	300 (150, 850)	1, 1900	0.27
Pony – other	14	265 (200, 453)	1, 750	1.00
Horse – other	17	350 (290, 550)	1, 1750	0.48
Unknown	37	180 (100, 260)	1, 820	0.83
Total	304	235 (130, 400)	1, 2550	

Other – horse: Appaloosa (n = 6), Paint (n = 7), Arab (n = 2), Clydesdale (n = 1), Warmblood (n = 1); Other – pony: Brumby (n = 3), Hackney pony (n = 1), Miniature pony (n = 3), Welsh pony (n = 7). Excludes – five horses and ponies that were passed in, one each of the following breeds: thoroughbred, quarter horse, stock horse, Pony – other and Horse – other; one pony (Shetland) that was unsold; and two horses and ponies that were sold to an unknown purchaser.

^a^ Differences within breeds were assessed using ANOVA. Tukey's Honestly Significant Difference test was used to compare breeds with the stated reference.

## Discussion

Although there is considerable media attention and publicity surrounding sale of thoroughbreds through public auctions,^1^, ^2^, ^8^, ^9^ only 16% of horses and ponies sold at the Pakenham sale in the study period were thoroughbreds. While the incidence of thoroughbreds attending sales is likely to vary from location to location, the saleyard in this study was chosen because was the closest to metropolitan Melbourne where the largest concentration of racing thoroughbreds in Victoria reside, with large racing centres at Pakenham, Flemington, Cranbourne and Caulfield tracks. Crossbred or mixed breed horses and ponies were the largest (30%) breed group presented at the sales, followed by thoroughbreds, quarter horses and standardbreds. These breeds attended the sales in similar proportions to breed representations reported in previous research reporting whole horse industry demographics,^14^, ^15^ yet there is little media attention paid to the fate of these horse breeds.

The breed distribution in this current study was consistent with previous research.^14^, ^15^ The proportion of thoroughbreds in previous studies has varied between 10% and 22%. Previous research on the number of horses in Australia, only reported thoroughbreds as those registered for racing and breeding at the time of the study but did not specify the number of thoroughbreds participating in the wider horse sector.^14^ The proportion of thoroughbreds in the current study population was slightly lower than in a previous Victorian study of breeds in the recreational horse sector which also found that the many of the thoroughbreds participating in the recreational horse sector had previous racing history.^15^ The reduced proportion of thoroughbreds in the current study may reflect a decline in the thoroughbred population over time, in line with the reduction in the size of the Australian foal crop, or that fewer thoroughbreds were being sent through the saleyards during the study period.^18^ While previous researchers have described the number of thoroughbreds within the racing and breeding industries or the recreational horse industry,^14^, ^15^ further research is needed to describe the number of horses and ponies that reside in Australia and the overall breed distribution of that population. The percentage of Australian stock horses (ASH) in this study (5%) was similar to those reported for the Victorian recreational horse industry study but lower than the 16% that has been estimated for the national horse population.^14^, ^15^ Data from the Australian Stock Horse Society shows that the number of ASH registered in Victoria was much less than those registered in New South Wales and Queensland,^19^ suggesting that the distribution of breeds varies between states in Australia.

The majority of horses and ponies sold at the Pakenham sales complex had a BCS of three or above (77%) and 76% had okay or good hoof condition. This suggests that these horses and ponies had been kept at an adequate level of husbandry prior to coming to the sales. Thoroughbred horses were in similar body condition and hoof condition to other horses and ponies at the sale. The BCSs coupled with hoof husbandry in this study showed that the majority of thoroughbreds were not in racing condition when they passed through Pakenham. These findings suggest that these thoroughbreds are not being sent to the sales directly after retiring from racing but were more likely coming from spelling or a post‐racing home. There was no difference in BCS between horses and ponies sold to private buyers and slaughter. This suggests that there are factors other than body mass and condition that influence the destination of horses from the sales.

The purchase price in this study was not a good predictor of purchaser type. The ponies sold in this study were more likely to be purchased by private buyers, horses were more likely to go to slaughter, despite horses being sold for a higher median price than ponies. This may be due to horses having a higher proportion of suitable body mass for use in meat compared to ponies, or other logistical factors such as the ability to transport safely. There is a perception that many horses sold through the saleyards are thoroughbreds and that the majority of these are sold to slaughter.^2^, ^3^, ^4^, ^5^, ^9^, ^11^, ^16^ Thoroughbreds were not the majority of the horses presented for sale in this study. There was also no significant difference between thoroughbreds, quarter horses, riding ponies or standardbreds in the likelihood of being purchased by slaughterhouse buyers. This is despite there being no significant difference in sale price between thoroughbreds and other breed groups with the exception of Crossbred horses. The similarity in price between these breeds may be unique to horses that attend mixed sales. A previous Australian study investigating horses advertised for sale in a magazine found that thoroughbreds were priced significantly lower than other breeds.^20^ This suggested that thoroughbreds were less desirable to private buyers, despite thoroughbreds having the largest breed representation in the recreational horse sector.^15^ There may be a perception that thoroughbred horses are leaving racing because they carry injuries and owners do not want to invest the time and money in healing them.^6^, ^21^ However, in recent studies the majority of thoroughbreds retired for performance reasons, with fewer than 30% due to injury. Nearly half of those injured horses were subsequently engaged in ridden activities.^22^, ^23^ There is little data available regarding the factors that influence the purchase of horses from mixed sales and this warrants further study.

The proportion of horses and ponies purchased by slaughterhouse buyers that were racing breeds (41%) in this study was lower than the 53% reported by previous research on horses going through a Queensland export abattoir.^10^ However, that study included animals that ‘looked like they were of thoroughbred type’, even though some were missing brands, and others had brands used by other breed associations as well as the Thoroughbred industry. Further research to describe all horse breeds that are being processed for meat in Australia with comparisons to the breed distributions of the horse population in Australia is needed.

Female horses were more likely than males to be purchased for slaughter and were sold for a lower median price. This may be due to the perception that mares can be moody or ill‐tempered making them potentially less desirable as a riding prospect.^24^ This perception may also limit their potential use to that of a broodmare, with little other value.^25^ It is possibly also an extension of our culture, where women still struggle to achieve full equality with their male counterparts. Riders draw on stereotypes of the sexes when making judgements about a horse's ability to perform in different disciplines.^24^ When asked to choose a sex for specific disciplines riders placed mares at the bottom choice in each of dressage, show jumping and trail riding disciplines.^24^ Despite mares routinely competing at high levels, the recent gold and silver medallists in individual freestyle dressage and the individual eventing gold medal winner at Tokyo Olympics 2021 all competed on mares. While there is no data on attitudes of people regarding horse sex when selecting horses for racing, it is interesting to note that three of the top racing horses in Australia over the last 20 years have been mares–Makybe Diva, Black Caviar and Winx.

While 64% of the entire males were ponies, the majority (72%) were from one vendor. Information provided by the vendor at the sale indicated that these ponies were from a deceased estate where the ponies had been left to breed and fend for themselves as the owner's health declined. This is likely to have biased the results on entire males and these results should therefore be interpreted with caution.

One of the limitations of the current study was that the overall results for this study were potentially skewed by a large number of ponies entered from one vendor at the December sale. Due to the limited sample size, comparisons between the full dataset with the dispersal ponies in it and a reduced set without the ponies were not undertaken. It is unclear from this study if the inclusion of a large intake of animals from one vendor was a singular event or a more frequent occurrence. Future longitudinal studies are required to investigate homogeneity of the population attending sales.

This pilot study provides valuable insight into the horses attending the mixed breed sales in Victoria. However, features unique to this saleyard may limit the application of results in other types of sales and sales in other locations. Further research is needed to better understand the demographic information and condition of horses attending sales throughout Australia and how this compares to the general horse population to identify risks for sale attendance and differences in outcomes when purchased at sale. Further research is needed to investigate the decision‐making process for people purchasing horses through a sale.

With public concerns around the overbreeding of horses, the finding that the largest breed group was crossbreds warrants further investigation into the factors that influence the decision to breed and the intended uses and markets for the horses that are being produced.

## Conflicts of interest and sources of funding

There are no sources of funding to declare for this study. The authors have read the journal's policy and have the following competing interests to declare: Racing Victoria Ltd. provided support for MLF in the form of a thesis stipend as part of a research contract (Racing Vic 045083 15/16) to conduct a separate project. AgriFutures provides support to MLF and JRG to work on another unrelated project. These external funders had no role in the design of the study; in the collection, analyses, or interpretation of data; in the writing of the manuscript, or in the decision to publish the results. There are no patents, products in development or marketed products associated with this research to declare.
